# An open chat with…Pierre Santucci: a testimonial on why we should engage with scientific societies and their journals

**DOI:** 10.1002/2211-5463.70045

**Published:** 2025-04-25

**Authors:** Pierre Santucci, Ioannis Tsagakis

**Affiliations:** ^1^ Aix Marseille Univ, CNRS, LISM, IMM FR3479, IM2B Marseille France; ^2^ FEBS Open Bio Editorial Office Cambridge UK

**Keywords:** early career researcher, FEBS, mycobacteria, not‐for‐profit, scientific societies

## Abstract

Scientific societies constitute a cornerstone in our scientific landscape by supporting research activities, fostering networking opportunities and promoting extensive training and education in basic sciences. As membership‐based organisations, scientific societies provide their members with access to meetings, workshops, educational programs and also funding opportunities through fellowships and awards. Societies also run and supervise the publishing activities of their sponsored journals that benefit authors and the entire community by enabling the publication of high‐quality studies by scientists, for scientists. Most societies or not‐for‐profit publishing organisations financially lean on memberships and their journals to support their activities. Therefore, engaging with and advocating for scientific societies and not‐for‐profit journals is important to help sustain their impactful activities and to raise awareness for fellow unaware scientists. In this piece, we discuss with our newly appointed Publishing Liaison Officer, Pierre Santucci, how scientific societies and sponsored journals can have a direct and beneficial impact on a scientist's career. Pierre also provides a short testimonial of his experience with FEBS initiatives and FEBS Press journals.

## Graphical abstract

Pierre SANTUCCI is a principal investigator at the CNRS—Aix Marseille Université and recipient of a FEBS Excellence Award. He was also recently appointed *FEBS Open Bio* Publishing Liaison Officer. In this *Open Chat with…*, he discusses the importance of engaging with scientific societies and their journals, and highlights through some of his personal experience how this engagement benefited his professional career.

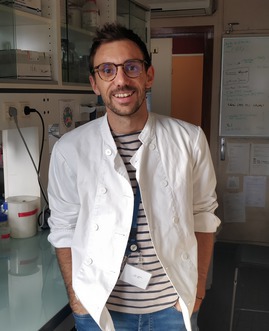

## Q: Do you think established scientists should inform Early Career Researchers (ECRs) about scientific societies and how can ECRs benefit from becoming a member?

**A**: First and foremost, if we wish for a system in which scientists engage more with societies, in order to gain a mutually beneficial relationship, we simply need to educate and inform our junior scientists about society and (re)create ‘a culture of the society’.

At the undergraduate levels, BSc or MSc students join research groups for their first internships and very often they are not aware that scientific societies exist and what their primary activities are; to support science. Hence, it is the principal investigator’s (PI) responsibility to spread the words and talk to the next generation of scientists as early as possible about the multiple advantages of scientific societies.

In my case, I was fortunate enough to be introduced very early to the concept of ‘a society’ by my PhD supervisor, Stéphane Canaan. Indeed, in 2015 I was only 22 years old when during my MSc internship in his lab, I got my first membership to the French Society for Microbiology (SFM) which is part of the Federation of European Microbiological Societies (FEMS). With this membership, I was able to attend the SFM‐sponsored 4th Edition of the French Working Group Symposium on Mycobacteria, ‘The MycoClub’. This meeting had close to 200 participants and I spent over 2 days discussing hard‐core mycobacteriology. I was blown‐away by how friendly the atmosphere was. This is where I discovered that, not only was I becoming part of a community, but also that my membership at the SFM enabled me access a large number of webinars, books, training and other scientific meeting opportunities.

There is absolutely no doubt that one of the most beautiful aspect of science resides in its interdisciplinarity. Hence, I was being trained as a microbiologist but would definitely use different techniques and approaches that belong to alternative scientific fields, including genetics, molecular biology, biochemistry or microscopy for example. This is where I have started to realise that there are actually many societies that you can identify with and subsequently become a member of. Importantly, each society can give you access to different opportunities and help you become part of a distinct community. This is where in 2018, I discovered the French Society of Molecular Biology and Biochemistry (SFBBM) and the Federation of European Biochemical Societies (FEBS), while submitting one of my PhD research articles to the *The FEBS Journal*.

It's a shame but it was actually quite late during my PhD training that I realised that some societies publish their own journals, and that the journals constitute a major source of income that is fully reinvested towards their members through meaningful activities. Such information is so important and may often be omitted when training the next generation of scientists.

Again, if we are to aim for a stronger ‘culture of the scientific societies’ in which more scientists believe and comply to it, we need to spread the word more widely. This ‘culture of the society’ that we currently are aiming for, should involve scientists at all levels, from the Head of Department, Professors and Lecturers, PIs, Staff scientists, post‐doctoral fellows, PhD candidates to MSc and BSc students that make their first step in the lab. Creating this culture where we share knowledge and personal experience by emphasising the key aspects of societies and their not‐for‐profit journals is the way to go.

## Q: What would you say are the perceived benefits or challenges associated with being a member of a scientific society?

**A**: In fact, a good way of highlighting how beneficial it is to become a member of one or multiple societies, is to first discuss the pros and cons and secondly to share with you my personal experience about it.

Joining a scientific society comes with a variety of benefits. Firstly, as mentioned earlier, you are part of a community composed of individuals that share your passion and dedication to a specific scientific field. Within this community, you can capitalise from a wide range of networking opportunities such as connecting with peers and mentors but also meet experts from outside academia through events, conferences, and meetings. Using this network, you have an almost unlimited way of engaging in new collaborative research projects and initiatives. This directly contributes to one's professional development, which can also be strengthened by benefiting from society‐sponsored workshops and training sessions to develop additional skills or being up‐to‐date with all the new technological and biological trends.

Being part of a society often facilitates access to sponsored, academic journals in many ways. This can be done, through meetings and events where you can showcase and discuss your work with professional editors that run the journal. Interacting with and publishing in society‐based journals is extremely rewarding as most of these journals are run by scientists for scientists. By working closely with the society and their members they offer a large panel of opportunities, from dedicated special issues, webinars or prizes for example.

This is also a major positive aspect; most societies recognise and help reaching scientific excellence through awards and fellowship application schemes. In order to be eligible, and get a well‐deserved recognition for scientific contributions and achievements in the field, one should be a member of the society.

Overall, the benefits are numerous, and such a non‐exhaustive list represents only a subset of them.

Now, let's list the cons…

There are none! Ok, maybe there is only one minor drawback that needs to be taken into consideration before joining the society, and it is the membership fees.

Most societies have fees and the cost of membership can vary widely depending on the society and the level of membership. However, most societies have extremely important discounts for students, postdocs and junior PIs under the age of 35 or 40 years old. Also, some additional schemes exist to facilitate the access from underrepresented countries with less resources.

In conclusion, I truly think that being part of a society – and making the most of it – is worth every penny. There are so many benefits that can be realised. More importantly, a large number of new members, assuming they have found the right society for them, will renew their memberships for years and years, therefore suggesting that the advantages are considerable.

## Q: What has been your experience with joining a scientific society?

**A**: I have introduced earlier some of the key advantages of being part of societies, but maybe highlighting how I personally benefited from my societies might help the next generation of scientists to better understand how proactively engaging with societies is beneficial.

I see my experience with scientific societies structured in three major phases in line with the emblematic quote of the US author, Alan Loy McGinnis, ‘Learn, Earn, Return; These are the 3 essential phases of life’.

Therefore, after initiating a shy but rich ‘Learn’ phase from 2015 to 2019; I have started to apply to multiple schemes coordinated by my societies and extensively gained from these applications. Indeed, my ‘Earn’ phase had started.

In 2019 just after completing my PhD, I applied to the SFM National PhD Thesis Award. My profile got shortlisted and I have been invited to present my work (and compete for the award!) at the annual SFM meeting in Paris. Luckily on that day, my work was selected by the jury among the other projects and candidates to be awarded with a national thesis prize in Microbiology from the French Society of Microbiology. In addition to the warm congratulations of everyone at the congress and my home institute, the award came with a monetary prize, a certificate, an article on the website of the SFM and a free membership for the upcoming year! That was fantastic!

This is where I realised that early career scientists need to apply and engage with their societies if they wish to be rewarded.

Moreover, I was also a new member of SFBBM and therefore FEBS; and at that time, I had just moved to London in the group of Maximiliano Gutierrez at the Francis Crick Institute. I was applying to multiple financial schemes in order to have my own postdoctoral fellowship to build up a more competitive CV. I knew about the competitiveness and prestige of the FEBS Long‐Term Fellowship Programme and decided to apply with Max' support, which had been very important all along this application process. Once again, I was fortunate enough to be amongst the selected ‘Fellows’. Despite the fact that this programme has been discontinued—yet replaced by the FEBS Excellence Award Programme—I can attest how good it was. The fellowship enabled covering a part of our salary, but also allowed us to connect with other Fellows through the annual Fellows Meeting and the annual FEBS Congress. I was part of the 2022 FEBS Fellows meeting in Vimeiro, Portugal that was held jointly with the 2022 IUBMB–FEBS–PABMB Young Scientists' Forum (YSF 2022) few days before the IUBMB‐FEBS‐PABMB Biochemistry Global Summit in Lisbon. Both venues were amazing, and it was a great opportunity for me to spend a few days with my peers, discuss science and get to know each other in a very friendly atmosphere. All FEBS Fellows were also kindly invited to contribute to *FEBS Open Bio* with Reviews or Research Protocols, which were highlighted in a dedicated Special Issue. That same year, my postdoctoral work was selected and awarded with the monthly prize called ‘Article of the Month’ organized by the SFBBM, one of the constituents of the FEBS.

In 2022, my postdoctoral work in the UK was coming to an end, and I was applying to go back to France to start my independent research group. My move back from the UK to France, was supported by a Long‐Term Fellowship of the European Respiratory Society (ERS), a not‐for‐profit international membership organisation that focuses on respiratory medicine, including pulmonary bacterial infections, my field of expertise. This undoubtedly helped me secure a very competitive, tenured, CNRS Research Associate Position this year.

Surprisingly, my appointment as junior PI did not stop my ‘Earn’ phase. In 2023, the SFBBM awarded me with a fully funded travel grant (for under 35 years old junior researcher) to attend the 47th Edition of the FEBS Congress in Tours.

The highlight of late 2023 was our junior group receiving the prestigious, newly launched FEBS Excellence Award—alongside outstanding group leaders from across Europe. This was mind‐blowing. Through this new scheme, FEBS supports junior PIs with €100 000 for research equipment and consumables over a 3‐year period. It also enables the laureates to attend the annual FEBS Excellence Awardee Meeting that precedes the annual FEBS Congress. Hence, in 2024 I met with the FEBS Excellence Awardees in Pavia and Milan. A wonderful experience to discuss hardcore biological concepts and new methodological approaches during our daily meetings but also very casually around some drinks while supporting our respective teams during the football world cup games every night. The good thing in Italy is that you can keep talking science until very late in the evening and laughing about it around an emblematic artisanal ‘gelato’. Again, another fantastic opportunity to connect with colleagues, get to know each other, share our work and eventually create new collaborations.

Finally, similarly to the 2022 FEBS Congress in Portugal, during this short stay in Italy, I also had the pleasure of meeting for the first time or reconnecting with some of the FEBS Press editors. What a blast I had sharing some ideas, preliminary data or complete manuscripts with professional editors that provide very constructive feedback and are very supportive. Based on this, our group has been invited to contribute to a Review for the FEBS Letters Excellence Awards Virtual Issue and actively discussed and planned the submission of two additional manuscripts in *FEBS Open Bio* and *FEBS Letters*.

All of this, was made possible by the FEBS and their sponsored journals, further emphasizing how beneficial they are for researchers.

## Q: Why is ‘giving back’ to the community and to scientific societies important and how does this benefit scientists?

**A**: What young scientists need to understand is that most of the societies are run by active scientists and that they do that *pro bono* in addition to their research and/or teaching duties. Without this active participation from our community, it's impossible for a not‐for‐profit scientific society to thrive or even survive. Therefore, engaging with various societies at different levels is critical.

In my case, I am perfectly aware of societies' contributions to my career, and giving back to my societies has become very important to me. In 2021, I officially joined the junior section of the SFM to help expand the network of junior microbiologists and promote the microbiology work performed by our talented junior scientists. In 2023, the ERS offered me to sit within the *ad‐hoc* panel of the ERS that awards the Short‐term and Long‐Term fellowships and since then I am part of the ERS College of Experts and contribute to the evaluation of the Fellowship applications of the ERS. In 2024, I have had the chance of being elected to sit within the administrative board of the SFBBM, and contribute to the running of our French society. Since 2025, I am a member of the committee of the *Article of the Months*, with some of the peers who awarded me this prize a few years ago.

Finally, I feel very grateful to FEBS for all the support me and my group have received over the years. I'm trying to return the favour as much as I can by engaging with FEBS and the FEBS Press journals. By closely working with the FEBS Excellence Awards and Fellowships Committee as well as Carolyn Ellis, former FEBS Communication Officer, we actively promote some of the laureates and their research activities though small articles within the FEBS Network. I was also invited to join a FEBS Open Bio‐sponsored Webinar: Supporting Early Career Researchers – what FEBS can offer. This session was meant to present some of the activities of FEBS and to encourage young scientists to participate and apply to multiple schemes offered by FEBS.

Finally, the best way to support FEBS, is to prioritize the publication of our articles within FEBS Press journals. Manuscripts are handled very efficiently by professional editors and active scientists, the feedback arising from the peer‐review process is always fair and constructive, and most importantly the revenue generated by the journals through the APCs or publishing agreements are directly reinvested within FEBS to support its the activities. Since 2022, our group has published five papers in FEBS Press journals and more are coming!

To conclude, supporting our societies can be achieved in many ways, from young students to well‐established PIs. Informing the new generation of scientists about societies, their actions and sponsored journals is key. Finally, if we want FEBS to sustain and last at least another 50 years, we must promote publication within FEBS Press, which remains the major source of funding for our not‐for profit society.

